# The Effect of Defect Morphology and Membrane Fixation on 3D Graft Material Displacement During Primary Wound Closure in Horizontal Bone Augmentation—An Ex Vivo Study

**DOI:** 10.1111/clr.70072

**Published:** 2025-11-05

**Authors:** Clemens Raabe, Emilio A. Cafferata, Wenjie Zhou, Katharina M. Müller, Neelam Lingwal, Ausra Ramanauskaite, Frank Schwarz, Emilio Couso‐Queiruga

**Affiliations:** ^1^ Department of Oral Surgery and Implantology Goethe University, Carolinum Frankfurt am Main Germany; ^2^ Department of Oral Surgery and Stomatology, School of Dental Medicine University of Bern Bern Switzerland; ^3^ Oral Peri‐Implant Research Group, School of Dentistry Universidad Científica del Sur Lima Peru

**Keywords:** alveolar ridge augmentation, bone substitutes, cone‐beam computed tomography, dental implants, grafts, guided bone regeneration, surgical flaps

## Abstract

**Objectives:**

This preclinical study evaluated the influence of two defect morphologies on graft material displacement (GMD) during primary wound closure in horizontal bone augmentation (HBA). Secondary aims included assessing the effect of membrane stabilization and the role of local soft tissue characteristics on GMD.

**Materials and Methods:**

Standardized HBA procedures following guided bone regeneration principles were performed on fresh pig hemimandibles. Each mandible received two sequential HBAs, randomized for defect morphology—partially contained (PCD) vs. contained (CD)—and membrane stabilization with (+Pins) or without (−Pins) four fixation pins. GMD was assessed using cone‐beam computed tomography and intraoral scanning by comparing graft dimensions before and after wound closure, at nine levels from the implant platform and across nine delimited sections, respectively.

**Results:**

Sixty HBA procedures were analyzed. A notable GMD was observed for both PCD and CD, with no significant differences between them. In contrast, membrane stabilization significantly reduced three‐dimensional GMD across all sections, with the most pronounced effect in the central‐crestal section (*p* < 0.001). At the implant platform level, GMD was −15.8% ± 25.6% with pins vs. −38.1% ± 27.4% without pins (*p* < 0.001). Across all groups, GMD occurred in an apico‐lateral direction, with the greatest volume loss in the central‐crestal, mesial, and distal crestal sections. Soft tissue phenotype did not affect GMD (*p* ≥ 0.240).

**Conclusion:**

Defect morphology did not significantly influence the notable apico‐lateral GMD. However, membrane stabilization using pins effectively reduced graft displacement, minimizing movement during primary wound closure.

## 1 Introduction

Tooth extraction initiates physiological wound healing processes that ultimately result in varying degrees of alveolar ridge deficiencies (Couso‐Queiruga et al. 2021). Alveolar ridge defects can be classified as vertical and/or horizontal, with horizontal deficiencies further subdivided into contained and non‐contained types, based on their potential to stabilize graft materials (Benic and Hämmerle 2014; Buser et al. 2022). These dimensional alterations can pose challenges for dental implant therapy for tooth replacement, often necessitating bone augmentation procedures either prior to or simultaneously with implant placement (Couso‐Queiruga et al. 2022; Raabe et al. 2024). Successful bone regeneration contributes to the satisfactory long‐term outcomes of dental implant therapy, and high willingness of patients to repeat the procedure if needed (Braun et al. 2025; Chappuis et al. 2017, 2018), with guided bone regeneration (GBR) being the most widely used and well‐documented biological principle for horizontal bone augmentation (HBA) (Benic and Hämmerle 2014).

GBR employs barrier membranes to selectively allow the migration of osteogenic cells into the defect area while excluding undesired cell types (Benic and Hämmerle 2014). Non‐resorbable polytetrafluoroethylene (PTFE) membranes reinforced with titanium provide superior structural support but are associated with higher rates of postoperative complications and necessitate a second surgical intervention for their removal (Leblebicioglu and Tatakis 2023). For this reason, resorbable membranes are often preferred in clinical practice (Buser et al. 2023). However, when used without additional support, barriers are prone to collapsing into the defect site, thereby limiting the volume of regenerated tissue. Thus, to prevent membrane collapse and maintain the intended regenerative space, the use of bone graft materials is generally recommended (Benic and Hämmerle 2014; Buser et al. 2023).

In this context, effective graft stabilization is crucial for successful healing, as micromovements of graft materials contribute to integration failure (Hämmerle et al. 2008). Fully contained defects, such as 4‐wall defects in fresh extraction sites with intact socket walls, are considered to provide ideal stability for graft materials. In contrast, resorption of the facial bone wall during early healing often transforms a 4‐wall defect into a 3‐wall defect with pronounced orofacial dimensions. In these cases, graft material stabilization relies primarily on the remaining adjacent bone walls, and membrane stabilization is typically not required (Benic and Hämmerle 2014; Buser et al. 2022; Chappuis et al. 2013). However, as healing progresses, ongoing remodeling of the alveolar ridge could lead to more extensive deficiencies, compromising the natural graft containment and potentially necessitating the use of additional measures to ensure adequate graft stabilization (Benic and Hämmerle 2014; Buser et al. 2022).

Although HBAs have demonstrated clinical effectiveness, recent studies highlight the risk of intraoperative graft material displacement (GMD) during flap manipulation for primary wound closure. Notably, particulate grafts covered with collagen membranes are susceptible to significant collapse, particularly at the crestal level of the grafted area (Mir‐Mari et al. 2016). The extent of this collapse varies depending on grafting technique, material properties, and flap advancement technique (Mir‐Mari et al. 2016, 2017; Naenni et al. 2019; Raabe et al. 2025; Zhou et al. 2025). Nevertheless, the specific impact of defect morphology on GMD, as well as optimal strategies for maintaining graft stability, remains underexplored (Park et al. 2022).

Therefore, this preclinical study aimed to evaluate the effect of two distinct defect morphologies on GMD during primary wound closure in the context of HBA. The secondary objectives included assessing the effect of membrane stabilization with four fixation pins and the influence of local soft tissue characteristics on GMD. The null hypotheses were that defect morphology (H01), membrane stabilization (H02), and soft tissue phenotypical characteristics (H03) would not affect GMD.

## 2 Materials and Methods

This preclinical study was conducted at the Department of Oral Surgery and Implantology, Goethe University, Carolinum, Frankfurt am Main, Germany, between October and November 2024 and adheres to the ARRIVE guidelines 2.0. Since the study used fresh hemi‐mandibles from 30‐week‐old pigs (*n* = 30), obtained from a local butcher, ethics approval was not required. Before surgery, soft‐tissue phenotypic characteristics, such as the width of keratinized mucosa (KMW) and flap thickness (FT), were recorded using a PCPUNC15‐probe and an endodontic file. Flap thickness was measured at 3, 6, and 9 mm apically from the gingival margin.

### 2.1 Surgical Procedure

The hemimandibles were randomly allocated to one of two standardized horizontal bone defects, which were created at the second premolar sites. For each hemimandible, two HBA procedures were performed sequentially, randomized for membrane fixation.

**FIGURE 1:**
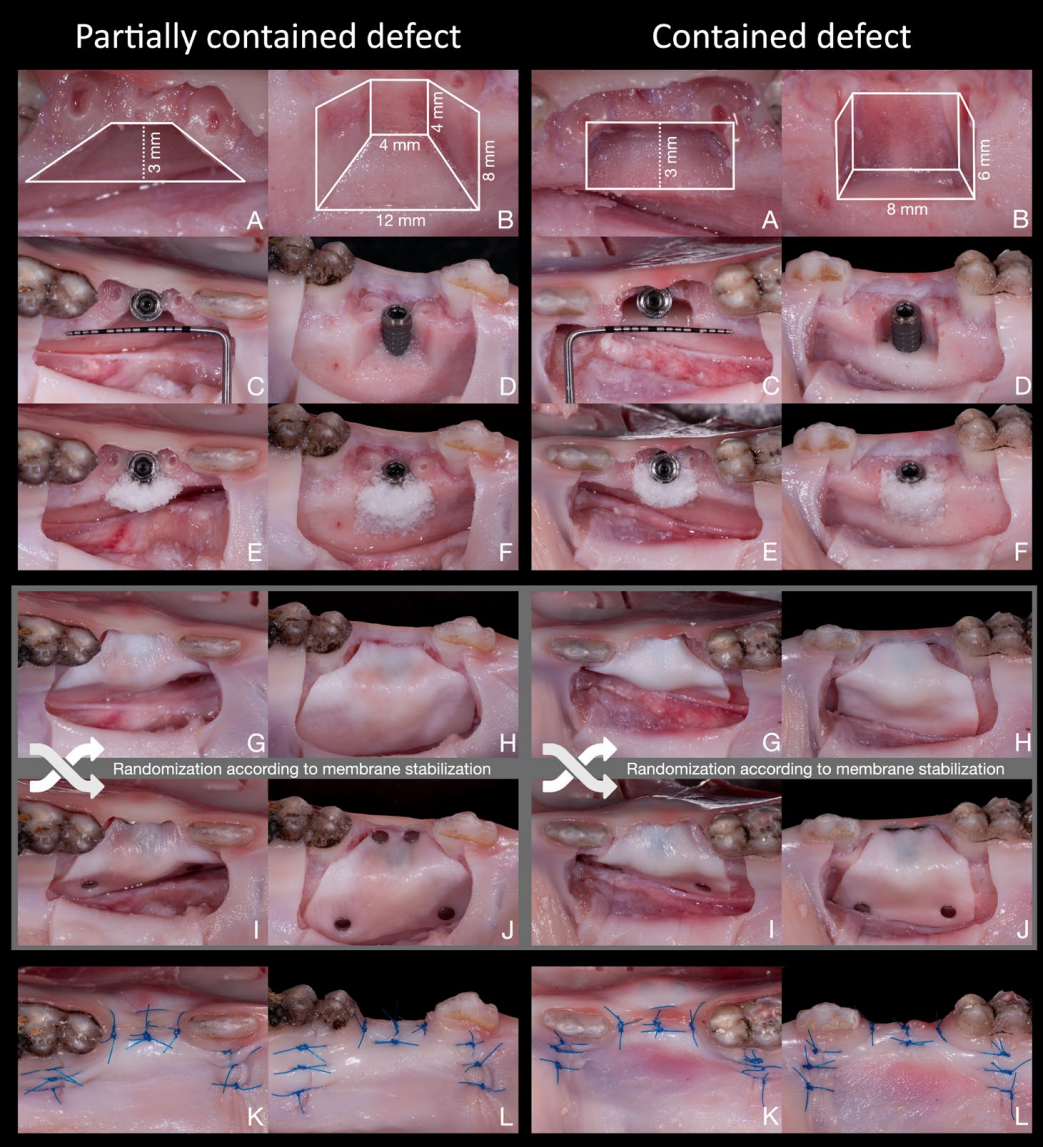
The clinical procedure demonstrates two distinct defect morphologies from occlusal and lateral perspectives (A, B). Defects were created, and implants were positioned 1 mm within the bony housing (C, D). Subsequently, grafting with deproteinized bovine bone mineral was performed (E, F), followed by coverage with a collagen membrane. Sites were randomly assigned to either no membrane stabilization (G, H) or stabilization using four pins (I, J). Primary wound closure (K, L).

All surgical procedures were performed by a single, experienced, and board‐certified oral surgeon (C.R.) using a standardized trapezoidal full‐thickness flap design, incorporating a sulcular incision at the second premolar and its adjacent teeth, along with two vertical releasing incisions. The flap was bluntly elevated using a periosteal elevator and a modified periosteal releasing incision was performed at the flap base to allow sufficient, tension‐free flap advancement. This involved an incision depth of approximately 0.5 mm, followed by blunt stretching of the tissues (Hur et al. 2015). Following epicrestal decoronation of the second premolar, standardized bone defects were created in the alveolar ridge with the following dimensions (mesio‐distal width × apico‐coronal height × oro‐facial depth), each resulting in a defect volume of 144 mm^3^ (Figure 1):

1. Partially contained defect (PCD): trapezoidal‐shaped, 12/4 × 8/4 × 3 mm.
2. Contained defect (CD): box‐shaped, 8 × 6 × 3 mm.

Subsequently, a dental implant (SPI Element 4.0 × 12.5 mm MC, Thommen Medical, Grenchen, Switzerland) was placed in the defect following the manufacturer's guidelines, with the implant platform aligned to the lingual bone crest and the outer circumference of the implant positioned 1 mm within the bony envelope.

The defects were then augmented using a particulate xenograft (BioOss, Geistlich Pharma AG, Wolhusen, Switzerland) soaked in a radiocontrast agent (Gastrografin, Bayer AG, Leverkusen, Germany), and over‐contoured by 2 mm, resulting in an initial graft material thickness of 3 mm (GMT_0). Subsequently, a collagen membrane (BioGide, Geistlich Pharma AG, Wolhusen, Switzerland) with or without the use of four fixation pins (Frios Membrane Tacks, Dentsply Sirona, Charlotte, North Carolina, USA) was applied.

Finally, the design resulted in four study groups with equal sample sizes:

– Partially contained defect, without membrane stabilization (PCD–Pins)
– Partially contained defect, with membrane stabilization using four pins (PCD + Pins)
– Contained defect, without membrane stabilization (CD–Pins)
– Contained defect, with membrane stabilization using four pins (CD + Pins)

An intra‐oral (IOS_1) (Trios 5 wireless, 3Shape, Copenhagen, Denmark) and Cone‐Beam Computed Tomography (CBCT) scan (CBCT_1, PaX‐Reve3D, Vatech, Hwaseong‐si, South Korea) of the “open‐wound” situation was taken. A blinded, experienced clinician (E.A.C) performed the standardized primary wound closure by using one horizontal mattress suture and nine single interrupted sutures (Optilene 5‐0, B. Braun, Melsungen, Germany 5‐0), with a spring dynamometer (Präzisionsdynamometer, 3B Scientific GmbH, Hamburg, Germany) verifying that the flap tension was ≤ 0.1 N during flap adaptation.

After wound closure, a second CBCT (CBCT_2) was obtained using the baseline exposure parameters. Subsequently, the sutures were gently removed and the mucoperiosteal flap was carefully reflected, without disturbing the collagen membrane. Then, a second intra‐oral scan (IOS_2) of the “re‐opened‐wound” situation was taken. Finally, all graft materials were removed, the site was thoroughly rinsed with water, and a second HBA procedure was conducted, alternating the use of membrane fixation from the first procedure.

### 2.2 CBCT Analysis

**FIGURE 2:**
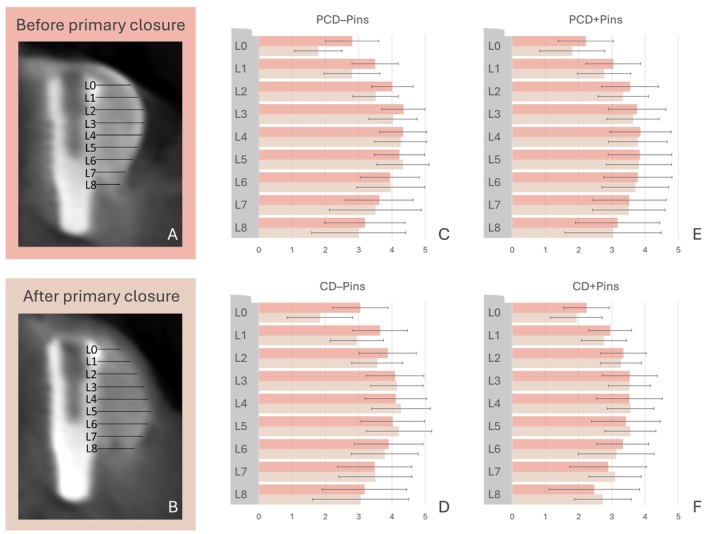
Cross‐sectional cone‐beam computed tomography (CBCT) reconstructions from the PCD − Pin group, captured before (A) and after (B) primary wound closure, depicting measurements at various apico‐crestal levels (L0–L8). Corresponding bar plots (±SD) present the mean graft material thickness (GMT) at these levels for the PCD/CD − Pin (C, D) and PCD/CD + Pin (E, F) groups, both before and after closure. CD, contained defect; GMT, graft material thickness; PCD, partially contained defect.

The CBCT datasets were analyzed using specialized software (byzz nxt, version 10.2.121, orangedental, Biberach, Germany). Cross‐sectional images were evaluated perpendicular to the mandibular curve and implant axis. Horizontal graft material thickness was measured before (GMT_1) and after primary wound closure (GMT_2) in 1 mm increments, starting from the implant platform and extending 8 mm apically (L0–L8, Figure 2). Changes in graft thickness reflecting GMD (mm/%) were calculated. All measurements were performed twice by a single calibrated investigator (W.Z) within an interval of 1 month to assess intra‐rater reliability.

### 2.3 IOS Analysis

**FIGURE 3:**
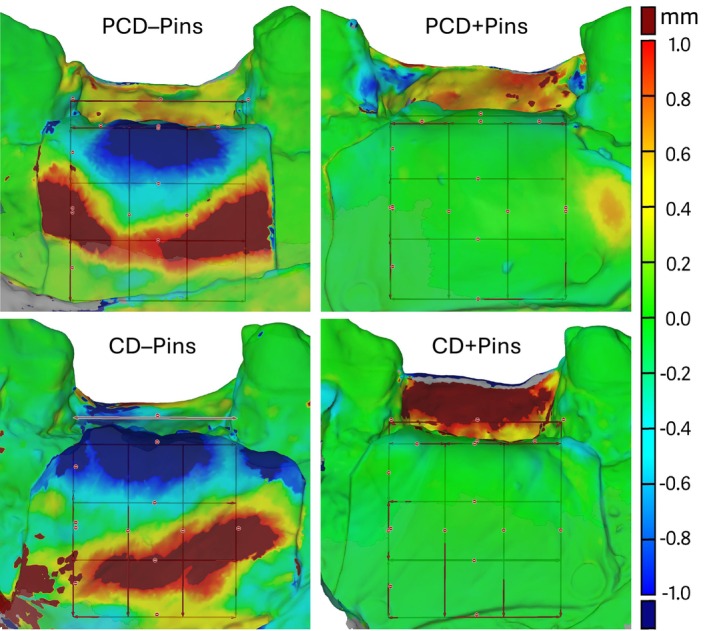
Volumetric analysis and visual representation of graft material displacement in a representative case from each group, based on the superimposition of intraoral scan data acquired before primary wound closure and after site reopening.

Three‐dimensional volumetric changes of the grafted region were assessed by superimposing intraoral scans taken before (IOS_1) and after (IOS_2) wound closure using specialized inspection software (Zeiss Inspect 2025, Carl Zeiss AG, Oberkochen, Germany). The alignment of scan data was semi‐automatically performed using a best‐fit algorithm, based on manually selecting the surface morphology of adjacent teeth to ensure consistency. IOS_1 served as the baseline reference, onto which IOS_2 was superimposed to visualize and assess post‐procedural dimensional changes. For the volumetric analysis, a standardized 12 × 12 mm region of interest (ROI) was centered on the grafted sites and aligned with the alveolar bone crest. This ROI was subdivided into a 3 × 3 grid, creating nine equal subsections (each 4 × 4 mm, Figure 3). The differences in surface levels between the IOS_1 and IOS_2 subsections were calculated and visualized as a color‐coded map, indicating both positive and negative deviations. For each 4 × 4 mm subsection, the arithmetic mean with its standard deviation of displacement values was computed to quantify localized volume changes.

### 2.4 Sample Size Calculation and Statistical Analysis

A sample size calculation for the prespecified effect (Cohen's *f* = 0.75) was carried out using the results of a previous project investigating GMD depending on flap advancement technique (Raabe et al. 2025). For a one‐way ANOVA with four groups, *α* = 0.05 and 90% power, the required total sample size was around 30 procedures (8 per group). Allowing for all pairwise post hoc comparisons increased the requirement to 42 (around 11 per group) for the primary endpoint. Therefore, we planned for 60 procedures (15 per group), which exceed both thresholds.

The intra‐rater reliability of the CBCT measurements was assessed by calculating the intra‐class correlation coefficient. The means of measurements between the two time points were used for the subsequent analysis. For comparisons between two groups, the Mann–Whitney *U* test was applied to non‐normally distributed data, while the *t*‐test was used for normally distributed data. For comparisons involving more than two groups, ANOVA or the Kruskal‐Wallis test was used depending on normality. For significant results, post hoc analysis was performed for *p*‐value adjustment, using Tukey's test for ANOVA and Dunn's test with a Bonferroni correction for the Kruskal–Wallis test. Additionally, a regression analysis was conducted to explore the relationship between the predictors (KMW, FT3, FT6, FT9, and FA2) and the dimensional changes. Descriptive statistics (minimum, Q1, median, mean, Q3, and maximum) were calculated. All analyses were performed in R (R version 4.2.3).

## 3 Results

A total of 60 HBA procedures were performed, equally randomized across 30 pig hemimandibles (*n* = 15 PCD − Pins, *n* = 15 CD − Pins, *n* = 15 PCD + Pins, *n* = 15 CD + Pins). The intra‐class correlation coefficient (ICC) for the linear metric CBCT measurements was 0.91, indicating a high degree of reliability in the examiner's measurements over time.

### 3.1 Defect Morphology

In the CBCT analysis, the overall mean relative GMD for PCD versus CD was −28.3% ± 28.2% vs. −28.6% ± 33.2% at L0, −14.8% ± 15.6% vs. −13.5% ± 19.3% at L1, and −9.4% ± 13.9% vs. −5.4% ± 14.9% at L2, respectively. No statistically significant differences in GMD were observed between PCD and CD across the different assessment levels (*p* ≥ 0.068). The corresponding GMT_1 and GMT_2 values (mm) for PCD versus CD were 2.5 ± 0.9 and 1.8 ± 0.8 vs. 2.6 ± 0.7 and 1.9 ± 0.7 at L0, 3.3 ± 0.8 and 2.8 ± 0.8 vs. 3.3 ± 0.6 and 2.9 ± 0.6 at L1, and 3.8 ± 0.7 and 3.4 ± 0.7 vs. 3.6 ± 0.6 and 3.4 ± 0.5 at L2, respectively. Descriptive statistics for GMT and GMD, along with the inter‐group comparison results analyzed using the Mann–Whitney *U* test, are presented in Tables 1, 2, S1 and Figure 2.

**TABLE 1:** Cross‐sectional cone‐beam computed tomography based analysis in the central aspect of the site. Descriptive statistics (means ± standard deviation) of the defect morphologies (PCD, CD) and modalities of membrane stabilization (+Pins, −Pins) at different apico‐crestal levels (L0–L8).

Group	Level	GMT_1 (mm)	GMT_2 (mm)	GMD (mm) (GMT1–GMT0)	GMD (%) (GMT1–GMT0)
PCD (PCD − Pins, PCD + Pins)	L0	2.5 ± 0.9	1.8 ± 0.8	−0.7 ± 0.7	−28.3 ± 28.2
	L1	3.3 ± 0.8	2.8 ± 0.8	−0.5 ± 0.5	−14.8 ± 15.6
	L2	3.8 ± 0.7	3.4 ± 0.7	−0.4 ± 0.5	−9.4 ± 13.9
	L3	4.1 ± 0.8	3.8 ± 0.8	−0.2 ± 0.7	−5.4 ± 16.7
	L4	4.1 ± 0.8	4.0 ± 0.8	−0.1 ± 0.9	−1.9 ± 20.8
	L5	4.0 ± 0.9	4.1 ± 0.9	0.0 ± 0.8	0.9 ± 19.1
	L6	3.8 ± 0.9	3.8 ± 1.0	0.0 ± 1.1	1.3 ± 28.0
	L7	3.5 ± 1.1	3.6 ± 1.2	0.1 ± 1.4	1.8 ± 40.6
	L8	3.0 ± 1.2	3.2 ± 1.4	0.2 ± 1.5	5.0 ± 46.1
CD (CD − Pins, CD + Pins)	L0	2.6 ± 0.7	1.9 ± 0.7	−0.8 ± 0.9	−28.6 ± 33.2
	L1	3.3 ± 0.6	2.9 ± 0.6	−0.4 ± 0.6	−13.5 ± 19.3
	L2	3.6 ± 0.6	3.4 ± 0.5	−0.2 ± 0.5	−5.4 ± 14.9
	L3	3.8 ± 0.7	3.8 ± 0.6	−0.0 ± 0.5	0.7 ± 13.6
	L4	3.8 ± 0.9	3.9 ± 0.6	0.1 ± 0.6	2.5 ± 16.2
	L5	3.7 ± 0.7	3.9 ± 0.9	0.2 ± 0.8	4.3 ± 20.4
	L6	3.5 ± 0.7	3.6 ± 1.0	0.2 ± 0.9	4.6 ± 25.7
	L7	3.2 ± 0.8	3.3 ± 1.0	0.1 ± 1.0	3.6 ± 31.1
	L8	2.8 ± 1.0	2.9 ± 1.1	0.1 ± 1.1	2.5 ± 40.5
No Pins (PCD − Pins, CD − Pins)	L0	2.9 ± 0.7	1.8 ± 0.7	−1.1 ± 0.8	−38.1 ± 27.4
	L1	3.6 ± 0.6	2.9 ± 0.7	−0.7 ± 0.6	−19.6 ± 17.1
	L2	3.9 ± 0.6	3.5 ± 0.6	−0.4 ± 0.5	−10.4 ± 13.9
	L3	4.1 ± 0.6	4.2 ± 0.7	−0.1 ± 0.6	−3.1 ± 13.4
	L4	4.2 ± 0.7	4.3 ± 0.7	0.0 ± 0.7	0.9 ± −16.8
	L5	4.1 ± 0.8	4.3 ± 0.7	0.1 ± 0.9	3.6 ± −21.1
	L6	3.9 ± 0.9	3.9 ± 0.9	0.1 ± 1.2	1.9 ± 30.2
	L7	3.5 ± 0.9	3.6 ± 1.1	0.0 ± 1.5	1.4 ± 42.6
	L8	3.0 ± 1.0	3.2 ± 1.3	0.2 ± 1.5	4.8 ± 48.1
Pins (PCD + Pins, CD + Pins)	L0	2.2 ± 0.7	1.9 ± 0.7	−0.3 ± 0.6	−15.8 ± 25.6
	L1	3.0 ± 0.7	2.8 ± 0.7	−0.2 ± 0.5	−7.7 ± 15.9
	L2	3.4 ± 0.8	3.3 ± 0.7	−0.1 ± 0.5	−4.0 ± 13.8
	L3	3.6 ± 0.8	3.6 ± 0.7	−0.1 ± 0.5	−1.7 ± 13.7
	L4	3.7 ± 1.0	3.7 ± 0.8	0.0 ± 0.6	−0.6 ± 16.0
	L5	3.6 ± 1.0	3.7 ± 0.9	0.1 ± 0.6	1.4 ± 17.7
	L6	3.4 ± 1.1	3.6 ± 0.9	0.1 ± 0.8	4.0 ± 22.3
	L7	3.2 ± 1.1	3.3 ± 0.9	0.1 ± 0.9	3.1 ± 28.0
	L8	2.8 ± 1.3	2.9 ± 1.2	0.1 ± 1.1	2.2 ± 37.4
PCD − Pins	L0	2.8 ± 0.8	1.8 ± 0.7	−1.0 ± 0.6	−36.3 ± 26.3
	L1	3.5 ± 0.7	2.8 ± 0.7	−0.7 ± 0.6	−19.9 ± 16.8
	L2	4.0 ± 0.6	3.5 ± 0.7	−0.5 ± 0.6	−12.6 ± 14.0
	L3	4.3 ± 0.6	4.0 ± 0.7	−0.3 ± 0.6	−7.2 ± 13.4
	L4	4.3 ± 0.7	4.3 ± 0.8	0.1 ± 0.8	−1.8 ± 17.6
	L5	4.2 ± 0.8	4.3 ± 0.8	0.1 ± 0.8	2.5 ± 20.0
	L6	3.9 ± 0.9	4.0 ± 1.0	0.0 ± 1.3	0.7 ± 32.7
	L7	3.6 ± 1.0	3.6 ± 1.4	−0.1 ± 1.8	−3.2 ± 48.8
	L8	3.2 ± 1.2	3.0 ± 1.4	−0.2 ± 1.7	−5.9 ± 53.7
PCD + Pins	L0	2.2 ± 0.8	1.8 ± 1.0	−0.4 ± 0.5	−18.3 ± 24.3
	L1	3.0 ± 0.8	2.8 ± 0.8	−0.3 ± 0.4	−9.0 ± 14.4
	L2	3.5 ± 0.9	3.3 ± 0.8	−0.2 ± 0.4	−5.7 ± 12.0
	L3	3.8 ± 0.9	3.6 ± 0.8	−0.1 ± 0.5	−3.3 ± 12.2
	L4	3.9 ± 0.9	3.8 ± 0.9	−0.1 ± 0.6	−2.0 ± 15.7
	L5	3.8 ± 1.0	3.8 ± 1.0	0.0 ± 0.7	−0.9 ± 18.5
	L6	3.8 ± 1.0	3.7 ± 1.0	−0.1 ± 0.8	−1.9 ± 22.4
	L7	3.5 ± 1.1	3.5 ± 1.1	0.0 ± 1.1	−0.3 ± 31.4
	L8	3.1 ± 1.3	3.1 ± 1.4	0.1 ± 1.2	−4.1 ± 38.7
CD − Pins	L0	3.1 ± 0.7	1.8 ± 0.6	−1.2 ± 0.9	−39.8 ± 28.7
	L1	3.6 ± 0.6	2.9 ± 0.6	−0.7 ± 0.5	−19.2 ± 13.7
	L2	3.9 ± 0.6	3.6 ± 0.4	−0.3 ± 0.5	−8.2 ± 13.9
	L3	4.1 ± 0.7	4.1 ± 0.5	0.1 ± 0.5	1.3 ± 12.3
	L4	4.1 ± 0.7	4.3 ± 0.5	0.2 ± −0.7	3.8 ± 16.1
	L5	4.0 ± 0.8	4.2 ± 0.6	0.2 ± 0.9	4.6 ± 23.0
	L6	3.9 ± 0.7	3.8 ± 0.9	−0.1 ± 1.1	−3.0 ± 27.5
	L7	3.4 ± 0.8	3.4 ± 0.9	0.0 ± 1.3	0.6 ± 36.2
	L8	3.2 ± 0.9	3.1 ± 1.1	−0.1 ± 1.4	−3.7 ± 43.5
CD + Pins	L0	2.2 ± 0.7	1.9 ± 0.8	−0.2 ± 0.6	−13.2 ± 27.5
	L1	2.9 ± 0.6	2.8 ± 0.7	−0.1 ± 0.5	−6.3 ± 17.6
	L2	3.3 ± 0.7	3.3 ± 0.6	−0.1 ± 0.5	−2.1 ± 15.8
	L3	3.5 ± 0.8	3.5 ± 0.6	0.0 ± 0.5	−0.1 ± 15.5
	L4	3.5 ± 1.0	3.6 ± 0.7	0.0 ± 0.6	0.9 ± 16.7
	L5	3.4 ± 1.0	3.5 ± 0.8	0.1 ± 0.6	3.8 ± 17.0
	L6	3.2 ± 0.8	3.1 ± 1.0	−0.2 ± 0.7	−6.0 ± 20.9
	L7	2.9 ± 1.2	3.1 ± 0.8	0.2 ± 0.7	7.3 ± 22.5
	L8	2.5 ± 1.4	2.7 ± 0.9	0.3 ± 0.9	10.4 ± 34.5

Abbreviations: CD, contained defect; GMT, graft material thickness; PCD, partially contained defect.

**TABLE 2:** Results of the Kruskal–Wallis test comparing the graft displacement depending on the defect morphologies (PCD, CD) and modalities of membrane stabilization (+Pins, −Pins) at different apico‐crestal levels (L0–L8) in the CBCT.

Level	PCD (±Pins) vs. CD (±Pins)	No Pins (PCD + CD) vs. Pins (PCD + CD)	CD + Pins vs. CD − Pins	PCD + Pins vs. PCD − Pins	CD + Pins vs. PCD + Pins	CD − Pins vs. PCD − Pins
L0	0.923	< 0.001*	0.016*	0.046*	1.000	1.000
L1	0.534	< 0.001*	0.098	0.065	1.000	1.000
L2	0.451	0.007*	0.427	0.271	1.000	1.000
L3	0.068	0.745	1.000	1.000	1.000	1.000
L4	0.173	0.160	1.000	1.000	1.000	1.000
L5	0.314	0.141	1.000	1.000	1.000	1.000
L6	0.865	0.399	1.000	1.000	1.000	1.000
L7	0.953	0.363	1.000	1.000	1.000	1.000
L8	0.994	0.750	1.000	1.000	1.000	1.000

Abbreviations: CD, contained defect; PCD, partially defect.

* *p*‐value < 0.05.

### 3.2 Membrane Fixation

In the CBCT analysis, the overall mean relative GMD was significantly smaller for Pins vs. no Pins, with −15.8% ± 25.6% vs. −38.1% ± 27.4% at L0, −7.7% ± 15.9% (*p* < 0.001) vs. −19.6% ± 17.1% at L1 (*p* < 0.001), and −4.0% ± −13.8% vs. −10.4% ± 13.9% at L2 (*p* = 0.007), respectively. No statistically significant differences in GMD were observed between PCD and CD across the assessment levels L3–L8 (*p* ≥ 0.141). The corresponding GMT_1 and GMT_2 values (mm) for Pins versus no Pins were 2.2 ± 0.7 and 1.9 ± 0.7 vs. 2.9 ± 0.7 and 1.8 ± 0.7 at L0, 3.0 ± 0.7 and 2.8 ± 0.7 vs. 3.6 ± 0.6 and 2.9 ± 0.7 at L1, and 3.4 ± 0.8 and 3.3 ± 0.7 vs. 3.9 ± 0.6 and 3.5 ± 0.6 at L2, respectively. Descriptive statistics for GMT and GMD, along with the inter‐group comparison results analyzed using the Mann–Whitney *U* test, are presented in Tables 1, 2, S1, and Figure 2.

### 3.3 Interaction Term Analysis

Within the subgroups, significantly less mean relative GMD was observed at L0 for CD + Pins vs. CD − Pins (−13.2% ± 27.5% vs. −39.8% ± 28.7%, *p* = 0.016), and PCD + Pins vs. PCD − Pins (−18.3% ± 24.3% vs. −36.3% ± 26.3%, *p* = 0.046). In contrast, no significant differences were found when comparing the two defect morphologies based on membrane stabilization or when analyzing the two membrane stabilization methods within each defect morphology at any other level (Table 2).

### 3.4 Volumetric Graft Material Displacement

**FIGURE 4:**
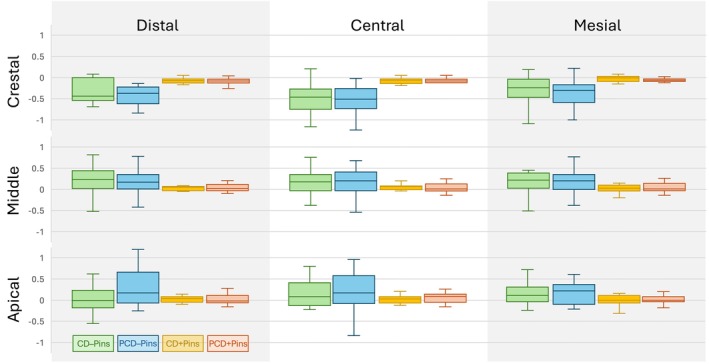
Intraoral scan‐based analysis within the nine areas of the grafted site. Boxplots depicting the mean graft displacement (mm) according to defect morphologies (PCD, CD) and modalities of membrane stabilization (+Pins, −Pins). CD, contained defect; PCD, partially contained defect.

The IOS analysis of the nine subsections revealed that GMD predominantly occurred in an apico‐lateral direction, with the greatest loss consistently observed in the central‐crestal section across all study groups, followed by the mesial and distal crestal sections. In contrast, volume gains were noted in the middle and apical sections in all groups (Figure 4). With regard to defect morphology, no statistically significant differences in GMD were found between PCD and CD across the nine sections (*p* ≥ 0.086). In terms of membrane stabilization, GMD was significantly lower in all sections when using Pins compared to no Pins (*p* ≤ 0.040), except for the apical‐distal section. Within the subgroups, a significantly lower mean relative GMD was observed in the three crestal sections for CD + Pins compared to CD – Pins, and for PCD + Pins compared to PCD – Pins (*p* ≤ 0.031). Visual representations of volumetric GMD in representative cases are shown in Figure 3. Descriptive statistics on GMD and inter‐group comparison results, are presented in Tables 3, 4, and Figure 4.

**TABLE 3:** Intraoral scan‐based analysis within the nine areas of the grafted site. Descriptive statistics (means ± standard deviation) of the graft displacement according to defect morphologies (PCD, CD) and modalities of membrane stabilization (+Pins, −Pins).

Graft displacement	Crestal distal (mm)	Crestal central (mm)	Crestal mesial (mm)	Mid distal (mm)	Mid central (mm)	Mid mesial (mm)	Apical distal (mm)	Apical central (mm)	Apical mesial (mm)
PCD (*n* = 30) (PCD − Pins, PCD + Pins)	−0.28 ± 0.31	−0.32 ± 0.36	−0.24 ± 0.28	0.14 ± 0.27	0.10 ± 0.25	0.13 ± 0.26	0.14 ± 0.34	0.14 ± 0.34	0.10 ± 0.21
CD (*n* = 30) (CD − Pins, CD + Pins)	−0.21 ± 0.23	−0.30 ± 0.33	−0.17 ± 0.26	0.14 ± 0.29	0.11 ± 0.21	0.12 ± 0.28	0.10 ± 0.23	0.08 ± 0.24	0.09 ± 0.22
No Pins (*n* = 30) (PCD − Pins, CD − Pins)	−0.39 ± 0.30	−0.52 ± 0.35	−0.34 ± 0.32	0.23 ± 0.35	0.15 ± 0.30	0.21 ± 0.35	0.13 ± 0.39	0.19 ± 0.38	0.17 ± 0.26
Pins (*n* = 30) (PCD + Pins, CD + Pins)	−0.10 ± 0.13	−0.10 ± 0.16	−0.07 ± 0.11	0.05 ± 0.13	0.05 ± 0.11	0.03 ± 0.10	0.01 ± 0.11	0.02 ± 0.13	0.01 ± 0.11
PCD − Pins (*n* = 15)	−0.47 ± 0.34	−0.56 ± 0.38	−0.39 ± 0.31	0.23 ± 0.35	0.12 ± 0.34	0.20 ± 0.31	0.26 ± 0.43	0.23 ± 0.45	0.18 ± 0.26
PCD + Pins (*n* = 15)	−0.09 ± 0.10	−0.08 ± 0.09	−0.09 ± 0.11	0.04 ± 0.09	0.05 ± 0.11	0.04 ± 0.11	0.02 ± 0.13	0.05 ± 0.12	0.01 ± 0.10
CD − Pins (*n* = 15)	−0.32 ± 0.25	−0.48 ± 0.34	−0.27 ± 0.32	0.23 ± 0.36	0.17 ± 0.27	0.22 ± 0.37	0.20 ± 0.20	0.15 ± 0.30	0.17 ± 0.26
CD + Pins (*n* = 15)	−0.10 ± 0.16	−0.11 ± 0.20	−0.04 ± 0.12	0.05 ± 0.16	0.05 ± 0.10	0.02 ± 0.09	0.00 ± 0.10	0.00 ± 0.13	0.00 ± 0.12

Abbreviations: CD, contained defect; PCD, partially contained defect.

**TABLE 4:** Results of the Kruksal–Wallis test comparing the graft displacement depending on defect morphologies (PCD, CD) and modalities of membrane stabilization (+Pins, − Pins) analysis within the nine areas of the grafted site.

Graft displacement	Crestal distal	Crestal central	Crestal mesial	Mid distal	Mid central	Mid mesial	Apical distal	Apical central	Apical mesial
PCD (±Pins) vs CD (±Pins)	0.318	0.835	0.086	0.812	0.773	0.946	0.264	0.211	0.830
No Pins (PCD + CD) vs Pins (PCD + CD)	< 0.001*	< 0.001*	< 0.001*	0.005*	0.040*	0.002*	0.111	0.028*	0.002*
CD + Pins vs CD − Pins	0.031*	< 0.001*	0.014*	0.100	0.456	0.172	0.818	0.467	0.131
PCD + Pins vs PCD − Pins	< 0.001*	< 0.001*	0.005*	0.105	0.797	0.356	0.354	0.308	0.111
PCD + Pins vs CD + Pins	0.490	0.477	0.155	0.834	1.000	0.998	0.498	0.964	0.999
PCD − Pins vs CD − Pins	0.162	0.806	0.155	0.446	0.938	0.995	0.339	0.871	0.997

Abbreviations: CD, contained defect; PCD, partially defect.

* *p*‐value < 0.05.

### 3.5 Soft Tissue Characteristics

The mean KMW was 7.6 ± 1.1 mm, while the mean FT3, FT6, and FT9 were 1.2 ± 0.3 mm, 1.1 ± 0.4 mm, and 0.8 ± 0.2 mm, respectively. The mean FA was 8.7 ± 1.0 mm. No statistically significant associations between KMW, FT3, FT6, FT9, or FA, and GMD were observed (*p* ≥ 0.240). Table 5 provides descriptive statistics on the local soft tissue phenotypical characteristics for the two defect morphologies.

**TABLE 5:** Descriptive statistics (mean ± standard deviation) of the local soft tissue phenotype for the two defect morphologies are presented.

	KMW (mm)	FT3 (mm)	FT6 (mm)	FT9 (mm)	FA (mm)
PCD	7.8 ± 1.13	1.3 ± 0.3	1.1 ± 0.4	0.8 ± 0.3	8.5 ± 1.1
CD	7.4 ± 1.14	1.1 ± 0.3	1.1 ± 0.3	0.9 ± 0.2	8.8 ± 0.8

Abbreviations: CD, contained defect; FA, flap advancement following periosteal release; FT3/6/9, flap thickness measured at 3, 6, and 9 mm apical to the incision line; KMW, keratinized mucosa width; PCD, partially contained defect.

## 4 Discussion

This preclinical study revealed a notable apico‐lateral graft material displacement from the implant platform during primary wound closure in HBA procedures, regardless of whether the defect was partially contained or contained. This displacement was not significantly affected by the soft tissue characteristics. However, stabilizing the membrane with four pins significantly reduced GMD. Consequently, hypotheses H01 and H03 were rejected, whereas H02 remained supported.

In regenerative procedures for HBA, the role of defect morphology on the stability of locally applied graft materials remains a topic of ongoing debate. This study examined two standardized defect configurations, differentiated by their capacity to contain graft materials, and, to our knowledge, is the first study to present 3D volumetric data investigating the directionality of GMD. The contained defect model aims to simulate early implant placement scenarios, characterized by the absence of the facial bone wall, a three‐walled configuration, and a vertical stop at the defect base. In contrast, the partially contained defect could represent late implant placement scenarios with extensive hard tissue resorption, resulting in a morphology with divergent bone walls on all sides. Interestingly, despite these differences, no significant difference in GMD was observed between PCD and CD at the implant platform (−28.3% ± 4.2% and −28.5% ± 2.7%, respectively). This similarity may be attributed to the overcontouring of graft material beyond the bony envelope, a strategy intended to compensate for expected postoperative remodeling following HBA (Elnayef et al. 2018). The GMD values reported in this study align with the previously published range from −23% to −65% observed in studies investigating the GMD during primary wound closure, although focusing on factors other than defect morphology (Mertens et al. 2019; Mir‐Mari et al. 2016; Raabe et al. 2025).

Membrane stabilization with four pins significantly reduced GMD at the implant platform to −15.7% ± 16.1% in the pin group, compared to −38% ± 9.4% in the no pin group. Previous studies reported a GMD of −22.9% ± 21.2% for a two‐pin stabilization at the base of the defect and −14.2% ± 11.5% for periosteal suturing (Mir‐Mari et al. 2016; Raabe et al. 2025). Therefore, the use of four pins or periosteal suturing may be more effective in reducing GMD by stabilizing a larger area compared to two pins alone. Assuming a standardized baseline GMT_0 of 3 mm before membrane application and observing a GMT_2 of 1.7–1.9 mm after primary wound closure, the final graft thickness was comparable across all groups, regardless of defect type or membrane stabilization method. However, after membrane application but before wound closure, GMT_1 measured 2.9 mm in the no‐pin group and 2.2 mm in the pin group, suggesting pin placement slightly compressed the graft material through membrane tension. In contrast, in the no‐pin group, similar compression appeared to occur during flap closure, as GMT_2 was comparable across all groups following primary wound closure. These findings imply that graft compression occurs regardless of membrane stabilization, so clinicians should anticipate this when deliberately over‐augmenting the site to achieve specific final graft dimensions. Membrane fixation, however, shifts the timing of compression, allowing clinicians to reshape the graft after pin placement and thereby enhance procedural predictability. Nevertheless, non‐fixation groups may also benefit from controlled compression during graft placement.

Alternative strategies to reduce GMD during primary wound closure include the use of L‐shaped soft‐block bone substitutes, titanium‐reinforced membranes, or autogenous block grafts in HBA (Mertens et al. 2019; Mir‐Mari et al. 2017). The present study's findings, demonstrating comparable post‐wound closure GMT_2 values across all groups, align with those of a micro‐computed CT animal study, which found that membrane fixation in contained defects did not impact the final ridge volume achieved through HBA procedures (Park et al. 2022). However, membrane stabilization may offer additional benefits, such as visual control over graft shaping prior to flap closure. The need for overcontouring and precise control of graft dimensions is highlighted by the substantial dimensional reductions reported for HBA during healing, amounting to approximately 26% after 4 months and up to 42% within the first postoperative year (Jiang et al. 2018; Park et al. 2023). These reductions may result from graft material and clot resorption, soft tissue pressure, or displacement of graft materials under biomechanical influences (Arnal et al. 2022). Moreover, graft stabilization has been shown to enhance the expression of osteogenic factors and result in significantly greater new bone formation compared to sites without stabilization (An et al. 2022, Ashoka Sreeja et al. 2025).

In the present study, the implant‐centered cross‐sectional radiographic analysis was complemented by an additional surface scan assessment to better elucidate the directionality of GMD across the entire grafted site. The 3D analysis was based on intraoral scans rather than CBCT volume reconstructions, as CBCT imaging is unsuitable for accurate surface reconstruction due to metal artifacts in the region of interest, grayscale thresholding challenges, and limited spatial resolution. The volumetric analysis revealed that GMD occurred in an apico‐lateral direction, with the most pronounced loss observed in the central‐crestal section corresponding to the crestal portion of the implant. This was followed by the mesial and distal crestal sections, which are located lateral to the implant. Notably, these crestal regions are critical for grafting, as they influence esthetic outcomes by supporting the soft tissue and contribute to peri‐implant health through the maintenance of buccal bone wall thickness (Monje et al. 2019, 2023). The observed apico‐lateral displacement was significantly reduced in groups where four pins were used for membrane stabilization, underscoring the importance of adequate graft material fixation in achieving predictable regeneration of hard tissue dimensions. Discrepancies in GMD measurements between CBCT and IOS analysis can be explained by the fact that the CBCT measurements were only carried out in cross‐sectional planes at the central aspect of the implant, whilst the IOS analyses correspond to the mean displacement within surface sections of 4 × 4 mm each.

Interestingly, the phenotypic characteristics of the sites were not associated with GMD, regardless of defect morphology. This finding aligns with a previous study that investigated the effect of two flap advancement techniques on graft material displacement and found no confounding influence of various flap characteristics (Raabe et al. 2025). Therefore, it is hypothesized that the degree of flap advancement, which was standardized across groups, may play a decisive role in GMD, as insufficient advancement could lead to high flap tension and, consequently, increased forces on the augmented site. Flap tensions during primary wound closure higher than 0.1 N were also associated with significantly more wound dehiscences and should therefore be avoided in order to ensure successful integration of the graft materials (Burkhardt and Lang 2010; Garcia et al. 2018). It should be noted that in the present study, baseline soft tissue characteristics, including flap thickness, were measured preoperatively; however, surgical flap manipulation, including periosteal releasing, may have altered these properties throughout the procedures.

Although the ex vivo design of the present pig study has been demonstrated to be reproducible for assessing graft material stability (Mir‐Mari et al. 2016, 2017; Naenni et al. 2019), several limitations must be acknowledged, and the results should be interpreted with caution. First, the standardized mandibular defect morphologies used in this study do not fully reflect the diversity of clinical scenarios, where a wide variety of defect configurations exist and gravitational effects in the maxilla may result in different outcomes. Second, the ex vivo design does not replicate dynamic postoperative factors such as muscular activity, mechanical forces, tissue swelling, or healing, which may exert pressure or tension over the soft tissues and thereby influence GMD in clinical scenarios. Third, despite meticulous efforts to preserve biomaterial positioning during wound reopening for postoperative intraoral scan, slight positional shifts cannot be entirely ruled out in the volumetric analysis. This may have disproportionately affected sites without pin fixation, potentially introducing bias. Nevertheless, CBCT‐based reconstructions would also have been subject to several limitations. Fourth, although the procedures were randomized and performed sequentially to minimize interferences, applying two procedures within the same hemi‐mandible may have introduced intra‐subject correlation. Consequently, the assumption of statistical independence may not have been fully met, which could have affected the precision of our estimates. Finally, future studies in the context of bone augmentation procedures should investigate GMD in defect configurations involving vertical components and evaluate its extent under clinical conditions during the early postoperative phase. Additionally, further research is needed to determine whether GMD influences the integration and long‐term stability of graft materials.

## 5 Conclusions

Within the limitations of the present study, it can be concluded that GMD during primary wound closure in HBA procedures:

– Is not affected by the two defect morphologies investigated.
– Occurs predominantly in an apical‐lateral direction.
– Can be reduced by membrane fixation with four pins, improving control over graft positioning and compression. However, fixation does not guarantee superior final graft dimensions compared with careful non‐fixation techniques.
– Is not influenced by soft tissue phenotypical characteristics.

## Author Contributions

**Clemens Raabe:** conceptualization, investigation, funding acquisition, writing – original draft, methodology, visualization, writing – review and editing, validation, software, formal analysis, project administration, data curation, resources, supervision. **Emilio A. Cafferata:** investigation, writing – review and editing, methodology, formal analysis, data curation. **Wenjie Zhou:** investigation, methodology, writing – review and editing, formal analysis, data curation, software. **Katharina M. Müller:** data curation, formal analysis, software, methodology, writing – review and editing. **Neelam Lingwal:** methodology, data curation, formal analysis, validation. **Ausra Ramanauskaite:** writing – review and editing. **Frank Schwarz:** writing – review and editing, conceptualization, resources, formal analysis, data curation, project administration. **Emilio Couso‐Queiruga:** conceptualization, writing – review and editing, methodology.

## Ethics Statement

The authors have nothing to report.

## Conflicts of Interest

The authors declare no conflicts of interest.

## Supporting information


**Table S1:** Descriptive statistics from the cross‐sectional cone‐beam computed tomography based GMD analysis in the central aspect of the site.

## Data Availability

The data that support the findings of this study are available from the corresponding author upon reasonable request.
