# Supplementary material for: The Effect of Defect Morphology and Membrane Fixation on 3D Graft Material Displacement During Primary Wound Closure in Horizontal Bone Augmentation—An Ex Vivo Study
Source: Clin Oral Implants Res. 2025 Nov 5;37(3):314–26. doi: 10.1111/clr.70072 (PMC12975682; doi:10.1111/clr.70072)
Supplement: Supplementary file 1 — Table S1: Descriptive statistics from the cross‐sectional cone‐beam computed tomography based GMD analysis in the central aspect of the site. [file CLR-37-314-s001.docx]

| **Group** | **Level** | **GMD**  **Min**  (mm) | **GMD**  **Q1**  (mm) | **GMD**  **Median**  (mm) | **GMD**  **Mean**  (mm) | **GMD**  **Q3**  (mm) | **GMD**  **Max**  (mm) | **GMD**  **SD**  (mm) |
| --- | --- | --- | --- | --- | --- | --- | --- | --- |
| **PCD** | L0 | -2.5 | -1.1 | -0.7 | -0.7 | -0.2 | 0.6 | 0.7 |
| **(PCD-Pins, PCD+Pins)** | L1 | -1.5 | -0.9 | -0.4 | -0.5 | -0.2 | 1.0 | 0.5 |
|  | L2 | -1.7 | -0.6 | -0.3 | -0.4 | 0.0 | 0.7 | 0.5 |
|  | L3 | -1.6 | -0.3 | -0.1 | -0.2 | 0.1 | 0.5 | 0.7 |
|  | L4 | -2.2 | -0.1 | 0.1 | -0.1 | 0.3 | 0.9 | 0.9 |
|  | L5 | -2.4 | -0.2 | 0.1 | 0.0 | 0.5 | 1.3 | 0.8 |
|  | L6 | -3.5 | 0.0 | 0.3 | 0.1 | 0.7 | 1.2 | 1.1 |
|  | L7 | -5.1 | -0.4 | 0.2 | 0.1 | 0.8 | 2.1 | 1.4 |
|  | L8 | -5.1 | -0.7 | 0.2 | 0.2 | 0.8 | 1.5 | 1.5 |
|  |  |  |  |  |  |  |  |  |
| **CD** | L0 | -3.0 | -1.3 | -0.7 | -0.8 | -0.1 | 0.6 | 0.9 |
| **(CD-Pins, CD+Pins)** | L1 | -2.3 | -0.8 | -0.4 | -0.4 | -0.1 | 0.8 | 0.6 |
|  | L2 | -1.2 | -0.4 | -0.2 | -0.2 | 0.0 | 1.3 | 0.5 |
|  | L3 | -1.6 | -0.2 | 0.1 | 0.0 | 0.3 | 0.9 | 0.5 |
|  | L4 | -1.8 | -0.1 | 0.3 | 0.1 | 0.4 | 1.0 | 0.6 |
|  | L5 | -2.3 | 0.0 | 0.3 | 0.2 | 0.6 | 1.3 | 0.8 |
|  | L6 | -2.9 | -0.1 | 0.4 | 0.2 | 0.6 | 1.7 | 0.9 |
|  | L7 | -3.5 | -0.1 | 0.4 | 0.1 | 0.5 | 1.8 | 1.0 |
|  | L8 | -3.6 | -0.2 | 0.2 | 0.1 | 0.6 | 2.3 | 1.1 |
|  |  |  |  |  |  |  |  |  |
| **No Pins** | L0 | -3.0 | -1.7 | -0.9 | -1.1 | -0.6 | 0.0 | 0.8 |
| **(PCD-Pins, CD-Pins)** | L1 | -2.3 | -1.0 | -0.8 | -0.7 | -0.4 | 1.0 | 0.6 |
|  | L2 | -1.7 | -0.7 | -0.4 | -0.4 | -0.2 | 1.3 | 0.5 |
|  | L3 | -1.5 | -0.4 | 0.0 | -0.1 | 0.3 | 0.8 | 0.6 |
|  | L4 | -1.7 | 0.0 | 0.3 | 0.0 | 0.5 | 1.0 | 0.7 |
|  | L5 | -2.3 | 0.0 | 0.3 | 0.1 | 0.7 | 1.3 | 0.9 |
|  | L6 | -3.5 | 0.2 | 0.3 | 0.1 | 0.7 | 1.4 | 1.2 |
|  | L7 | -5.1 | -0.1 | 0.5 | 0.0 | 0.8 | 2.1 | 1.5 |
|  | L8 | -5.1 | -0.2 | 0.4 | 0.2 | 0.8 | 1.6 | 1.5 |
|  |  |  |  |  |  |  |  |  |
| **Pins** | L0 | -1.3 | -0.9 | -0.2 | -0.4 | 0.0 | 0.6 | 0.6 |
| **(PCD+Pins, CD+Pins)** | L1 | -1.3 | -0.5 | -0.1 | -0.2 | 0.1 | 0.8 | 0.5 |
|  | L2 | -1.4 | -0.3 | -0.1 | -0.1 | 0.1 | 0.8 | 0.5 |
|  | L3 | -1.6 | -0.2 | 0.0 | -0.1 | 0.2 | 0.9 | 0.5 |
|  | L4 | -2.2 | -0.1 | 0.1 | 0.0 | 0.3 | 0.9 | 0.6 |
|  | L5 | -2.4 | -0.1 | 0.1 | 0.1 | 0.4 | 0.8 | 0.6 |
|  | L6 | -2.6 | -0.1 | 0.2 | 0.1 | 0.6 | 1.7 | 0.8 |
|  | L7 | -2.9 | -0.2 | 0.2 | 0.1 | 0.5 | 1.8 | 0.9 |
|  | L8 | -3.1 | -0.3 | 0.1 | 0.1 | 0.5 | 2.3 | 1.1 |
|  |  |  |  |  |  |  |  |  |
| **PCD-Pins** | L0 | -2.5 | -1.5 | -0.9 | -1.0 | -0.5 | -0.1 | 0.6 |
|  | L1 | -1.5 | -1.1 | -0.9 | -0.7 | -0.4 | 1.0 | 0.6 |
|  | L2 | -1.7 | -0.8 | -0.5 | -0.5 | -0.2 | 0.7 | 0.6 |
|  | L3 | -1.5 | -0.6 | -0.2 | -0.3 | 0.1 | 0.5 | 0.6 |
|  | L4 | -1.7 | -0.2 | 0.0 | 0.1 | 0.4 | 0.9 | 0.8 |
|  | L5 | -1.5 | -0.1 | 0.2 | 0.1 | 0.7 | 1.3 | 0.8 |
|  | L6 | -3.5 | -0.3 | 0.3 | 0.0 | 0.9 | 1.2 | 1.3 |
|  | L7 | -5.1 | -0.8 | 0.5 | -0.1 | 1.0 | 2.1 | 1.8 |
|  | L8 | -5.1 | -0.5 | 0.4 | -0.2 | 0.9 | 1.4 | 1.7 |
|  |  |  |  |  |  |  |  |  |
| **PCD+Pins** | L0 | -1.3 | -0.9 | -0.3 | -0.4 | -0.1 | 0.6 | 0.5 |
|  | L1 | -1.2 | -0.5 | -0.2 | -0.3 | 0.0 | 0.3 | 0.4 |
|  | L2 | -1.4 | -0.4 | 0.0 | -0.2 | 0.1 | 0.2 | 0.4 |
|  | L3 | -1.6 | -0.2 | 0.0 | -0.1 | 0.1 | 0.3 | 0.5 |
|  | L4 | -2.2 | -0.1 | 0.1 | -0.1 | 0.2 | 0.4 | 0.6 |
|  | L5 | -2.4 | -0.2 | 0.1 | 0.0 | 0.3 | 0.7 | 0.7 |
|  | L6 | -2.6 | 0.0 | 0.2 | 0.1 | 0.5 | 1.0 | 0.8 |
|  | L7 | -2.9 | -0.1 | 0.2 | 0.0 | 0.6 | 1.4 | 1.1 |
|  | L8 | -3.1 | -0.5 | 0.2 | 0.1 | 0.5 | 1.5 | 1.2 |
|  |  |  |  |  |  |  |  |  |
| **CD-Pins** | L0 | -3.0 | -1.9 | -1.1 | -1.2 | -0.6 | 0.0 | 0.9 |
|  | L1 | -2.3 | -0.9 | -0.8 | -0.7 | -0.4 | 0.6 | 0.5 |
|  | L2 | -1.1 | -0.5 | -0.3 | -0.3 | -0.2 | 1.3 | 0.5 |
|  | L3 | -1.2 | -0.2 | 0.1 | 0.1 | 0.4 | 0.8 | 0.5 |
|  | L4 | -1.4 | 0.1 | 0.3 | 0.2 | 0.6 | 1.0 | 0.7 |
|  | L5 | -2.3 | 0.1 | 0.4 | 0.2 | 0.6 | 1.3 | 0.9 |
|  | L6 | -2.9 | 0.3 | 0.4 | -0.1 | 0.6 | 1.4 | 1.1 |
|  | L7 | -3.5 | 0.2 | 0.4 | 0.0 | 0.6 | 1.2 | 1.3 |
|  | L8 | -3.6 | -0.2 | 0.4 | -0.1 | 0.5 | 1.6 | 1.4 |
|  |  |  |  |  |  |  |  |  |
| **CD+Pins** | L0 | -1.3 | -0.8 | -0.1 | -0.2 | 0.1 | 0.6 | 0.6 |
|  | L1 | -1.3 | -0.4 | -0.1 | -0.1 | 0.0 | 0.8 | 0.5 |
|  | L2 | -1.2 | -0.2 | -0.1 | -0.1 | 0.2 | 0.8 | 0.5 |
|  | L3 | -1.6 | -0.2 | 0.1 | 0.0 | 0.2 | 0.9 | 0.5 |
|  | L4 | -1.8 | -0.1 | 0.1 | 0.0 | 0.3 | 0.9 | 0.6 |
|  | L5 | -1.6 | -0.1 | 0.1 | 0.1 | 0.6 | 0.8 | 0.6 |
|  | L6 | -0.9 | -0.2 | 0.4 | -0.2 | 0.6 | 1.7 | 0.7 |
|  | L7 | -0.6 | -0.3 | 0.2 | 0.2 | 0.4 | 1.8 | 0.7 |
|  | L8 | -0.7 | -0.3 | 0.1 | 0.3 | 0.5 | 2.3 | 0.9 |

Supplementary Table 1 Cross-sectional cone-beam computed tomography based GMD analysis in the central aspect of the site. Descriptive statistics including Minimum, Q1, Median, Mean, Q3, Maximund and standard deviation of the defect morphologies (PCD, CD) and modalities of membrane stabilization (+Pins, -Pins) at different apico-crestal levels (L0-L8). GMD: graft material displacement. PCD: partially contained defect. CD: contained defect.
